# Neurometabolic Remodeling in Chronic Hiv Infection: a Five-Year Follow-up Multi-Voxel Mrs Study

**DOI:** 10.1038/s41598-019-56330-0

**Published:** 2019-12-24

**Authors:** Jasmina Boban, Majda M. Thurnher, Snezana Brkic, Dajana Lendak, Vojislava Bugarski Ignjatovic, Aleksandar Todorovic, Dusko Kozic

**Affiliations:** 1 University of Novi Sad, Faculty of Medicine Novi Sad, Hajduk Veljkova 3, 21000 Novi Sad, Serbia; 2 Oncology Institute of Vojvodina, Center for Diagnostic Imaging, Put dr Goldmana 4, 21204 Sremska Kamenica, Serbia; 3 Medical University of Vienna, Department fo Biomedical Imaging and Image-guided Therapy, Waehringer Guertel 18-20, 1090 Vienna, Austria; 4 Clinical Center of Vojvodina, Clinic for Infectious Diseases, Hajduk Veljkova 1, 21000 Novi Sad, Serbia; 5 Clinical Center of Vojvodina, Clinic for Neurology, Hajduk Veljkova 1, 21000 Novi Sad, Serbia

**Keywords:** HIV infections, Translational research

## Abstract

There is a lack of data about the long-term follow-up changes in neurometabolic profile and neuropsychological performance of HIV-positive subjects under continuous antiretroviral therapy (cART). The aim of the study was to assess changes in neurometabolic profile in chronically-infected, HIV-positive subjects during a five-year follow-up period, using multi-voxel proton magnetic resonance spectroscopy (^1^H-MRS). Nineteen neurologically asymptomatic, aviremic, HIV-positive subjects, underwent multi-voxel 2D MRS on a 3 T MR unit and synchronous neurocognitive assessment in a five-year follow-up period. Twelve voxels were placed in prefrontal cortices, anterior and posterior cingulate gyrus, intraparietal sulci, and frontal centrum semiovale white matter, to identify peaks of N-acetyl-aspartate (NAA), creatine (Cr), choline (Cho), and myoinositol (mI). Ratios of NAA/Cr, NAA/Cho, NAA/mI, mI/Cr, and Cho/Cr were analyzed. Longitudinal differences in ratios and neurocognitive scores were tested with the Wilcoxon signed-rank-test. Statistical significance was set at p ≤ 0.004 significant, and 0.05 > p > 0.004 trending toward significance. A significant longitudinal increase in NAA/Cr ratio was observed in 5/12 voxels, while there was a trend toward significance in an additional three. The increase in Cho/Cr reached statistical significance in one voxel. Changes in the mI/Cr ratio demonstrated a significant increase in 4/12 voxels. A progressive increase in NAA/Cr, followed by better neurocognitive performance, may be an indicator of brain plasticity in the setting of chronic HIV-related neuronal injury. A progressive mI/Cr increase could be partly explained by glial proliferation due to functional compartment remodeling and partly attributable to insufficient control of persistent neuroinflammation by cART.

## Introduction

Several longitudinal studies have shown changes in the biochemical profile of the brain in human immunodeficiency virus (HIV) infection, depending on the phase of infection and the introduction of combination antiretroviral therapy (cART)^1,2^. Although no signs of neuronal injury can be detected on magnetic resonance imaging (MRI) and magnetic resonance spectroscopy (MRS) in the early phase of HIV transmission, there are already prominent signs of ongoing inflammation. This inflammation is reflected by elevated levels of myoinositol (mI), a marker of microglial proliferation, and choline (Cho), a marker of membrane metabolism and gliosis. Soon after the initiation of cART, in the absence of neuronal dysfunction, normalization of inflammation markers can occur in several weeks^1^. The initial increase in inflammation markers will decline after cART initiation, although they do not reach the level of healthy controls^3^. Further in the course of disease, a decline in N-acetyl aspartate (NAA), a neuronal marker, occurs and becomes the dominant finding in chronically infected HIV-patients^4^.

In the first MRS study performed in our institution on 110 subjects (60 HIV-positive patients and 50 age- and gender-matched controls), the results clearly showed that HIV-associated neurodegeneration affects the whole volume of the brain. Furthermore, the results of that study, as well as other, smaller studies, confirmed ongoing inflammation under cART^2^. The neurodegenerative process associated with long-standing, well-controlled HIV infection results in progressive neurocognitive dysfunction^5^. The explanation for this process is thought to be due to chronic neuroinflammation and persistent immune activation, driven by an uneradicated pool of HIV-particles^6^.

In this longitudinal study, our aim was to assess longitudinal changes in the neurometabolic profile of neurologically asymptomatic HIV-positive individuals treated with cART during the follow-up period of five years, using multi-voxel proton magnetic resonance spectroscopy (^1^H MRS). In addition, a relationship between longitudinal changes in neurocognitive performance and MRS findings was investigated.

## Methods

### Participants

Baseline neuroimaging was performed in 31 HIV-positive patients, of whom 22 patients underwent follow-up imaging after five years. Three patients were excluded from the study based on the exclusion criteria. An overall total of 19 chronically infected HIV+ male patients on cART, mean age 45.16 ± 11.47, were included in this institutional review board-approved study (Ethical Committee of the Faculty of Medicine Novi Sad). Plasma viral load remained undetectable during the follow-up period in all patients. Participants underwent standard magnetic resonance imaging (MRI) and an MRS study on a 3 T MR unit (Trio Tim, Siemens, Erlangen, Germany), in the follow-up period of five years (e.g., baseline MRS studies were performed in 2011 and follow-up studies in 2016). Demographic and basic clinical data for both time points (including the nadir CD4 T-cell count, the current count of CD4+ T-cells, and cART duration) are summarized in Table 1.

**Table 1:** Demographic and basic clinical data for the study participants at baseline and after five-year follow up.

Variable	N	Mean	SD	Min	Max	Percentiles		
						25^th^	50th (Median)	75^th^
Age (years)	19	45.16	11.47	25	66	34.00	43.00	56.00
Education (years)	19	12.21	3.39	4	16	10.00	12.00	16.00
Nadir CD4 count (cells/mL)	19	267.32	208.62	11	694	105.00	200.00	460.00
CPE	19	8.92	1.23	7	10	8.50	9.00	10.50
**CD4 count (cells/mL)**								
2011	19	558.58	296.280	210	1122	261.00	526.00	801.00
2016	19	731.06	283.781	347	1195	450.00	754.00	984.00
**cART (years)**								
2011	19	5.65	2.25	1.5	11	2.25	5	8.75
2016	19	10.65	2.25	6.5	16	7.25	10	13.75

Inclusion criteria for the follow-up study were: completion of two MR examinations and synchronous neuropsychological testing; chronic HIV infection at the baseline (over one year after transmission); stable on cART (over one year after initiation); no signs of manifest neurocognitive disorder at baseline; and a normal conventional MRI. Exclusion criteria were: detectable viral load over the follow-up period (>50 copies/mL), current or past confounding neurological disease (opportunistic infection, multiple sclerosis, vascular and non-vascular dementia, neurodegenerative disease); brain tumors; traumatic brain injury; major psychiatric disorders; cardiovascular diseases (hypertension, chronic occlusive carotid disease, ischemic vascular dementia); active abuse of narcotic drugs (according to the Diagnostic and Statistical Manual of Mental Disorders); and hepatitis B or C coinfection at the baseline and over the follow-up period.

**Figure 1:**
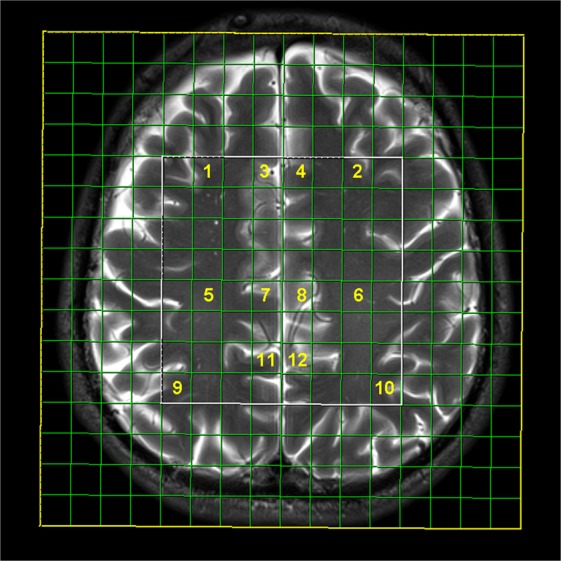
Multi-voxel network with analyzed voxel locations: prefrontal cortex in the right (1) and left (2) hemisphere; ventral anterior cingulate gyrus in the right (3) and left (4) hemisphere; dorsal anterior cingulate in the right (7) and left (8) hemisphere; centrum semiovale deep white matter in the right (5) and left (6) hemisphere; posterior cingulate gyrus in the right (11) and left (12) hemisphere; and intraparietal sulcus in the right (9) and left (10) hemisphere.

All selected patients were compliant with therapy, without any changes in the regimen of cART over the observed period of time. Three patients were excluded from follow-up imaging due to a change in the cART regimen (transient neuropsychiatric symptoms presented due to the introduction of efavirenz). A CONSORT study diagram showing patient selection in detail is shown in Fig. 1. For all subjects, the CNS penetrance efficacy score (CPE) was generated, with a mean value of 8.9 ± 1.3.

All subjects signed written, informed consent.

### Neuroimaging protocol

Imaging was performed in 2011 and 2016, using an eight-channel head array coil. The MRI protocol consisted of a three-planar, multi-sequential study: sagittal T1-weighted spin echo (repetition time 440 ms, time of echo 3.8 ms, thickness of the slice 5 mm); axial T2-weighted turbo spin echo (5150 ms, 105 ms, 5 mm); FLAIR (FLuid Attenuation Inversion Recovery) (8000 ms, 101 ms, 5 mm); and coronal T2-weighted turbo spin echo (7150 ms, 111 ms, 5 mm) MR tomograms.

Proton multi-voxel intermediate- (1700 ms, 135 ms) and short-echo-time (1700, 30 ms) MRS data sets were acquired using a two-dimensional (2D) PRESS (Point RESolved Spectroscopy) technique, with automatic volume-selective shimming. Features of the spectroscopic slab were: volume of interest 80 × 80 × 10 mm; thickness 10 mm; and region of interest positioned in the supracallosal white and gray matter. The multi-voxel network consisted of 64 voxels, of which we chose 12 symmetrical voxels in both hemispheres:

1, 2: the grey matter of the prefrontal cortex (PFC);

3, 4: the ventral part of the anterior cingulate gyrus (ACG);

5, 6: the frontal centrum semiovale white matter (FCSWM);

7, 8: the dorsal part of the ACG;

9, 10: the intraparietal sulcus (IPS); and

11, 12: the posterior cingulate gyrus (PCG) (Fig. 1).

Post-processing was performed on the Leonardo workstation (Siemens, Erlangen, Germany) by two experienced neuroradiologists. The post-processing protocol consisted of baseline corrections, peak identification, and metabolite ratio calculations.

In each subject, two experienced neuroradiologists in consensus, selected the voxels that best covered the primary regions of interest (ROI), based on their location on the matching images (high-resolution). The investigators were blinded to the time of acquisition of the data. The mean of the peak area for observed metabolites were computed form fitted spectral data.

The peaks identified were: NAA; Cho; mI; and total creatine (Cr), used as a reference marker. Ratios of NAA/Cr and Cho/Cr on intermediate-echo time and mI/Cr on short-echo time were analyzed.

### Neurocognitive assessment

Synchronous follow-up neurocognitive testing was performed at the same time points as MRS. All participants underwent a 90-minute neuropsychological test battery that assessed multiple neurocognitive functions typically found to be impaired in HIV-positive subjects. This battery consisted of: the Mini Mental Score Examination (MMSE)^7^; the Trial making test form A and B (TmtA, TmtB)^8^; the Rey auditory-verbal learning test (RAVLT)^9^; the Rey-Osterrieth complex figure test (ROCF)^10^; the Verbal fluency test-phonemic and categorical fluency tests^11^; the Verbal and visuospatial memory span^12^; the Wisconsin Sorting Card test^13^; and the Beck depression inventory scale II^14^. The same experienced clinical psychologist conducted all the neuropsychological testing. The study was conducted in compliance with the Helsinki Convention principles, was ethically approved, and every subject signed a written consent to participate in the research.

### Statistical analysis

Statistical analysis was performed using IBM SPSS software (version 21.0, Chicago, IL, USA). Descriptive statistics included mean values, standard deviation, median, minimum and maximum for continuous and median values, range (minimum-maximum), and interquartile range (IQR) for variables that did not follow a normal distribution.

Metabolite ratios of NAA/Cr, Cho/Cr, and mI/Cr that were obtained on the MRS scans in 2011 and in 2016 were compared using the Wilcoxon signed-rank test for repeated measurements. Due to the suspected instability of Cr, we performed longitudinal analysis calculations of NAA/Cho and NAA/mI ratios, in order to exclude the influence of Cr on metabolite ratios. The results of follow-up neurocognitive assessments performed at the same time as MRS were compared using the same test. We used this non-parametric test used because of the small sample size. For the same reason, we used Bonferroni corrections, designating p-values equal to or below 0.004 as significant (12 voxel locations, 0.05/12 = 0.004), and 0.05 > p > 0.004 as indicative of a trend toward significance.

### Ethical approval

All procedures performed in studies involving human participants were in accordance with the ethical standards of the institutional and/or national research committee and with the 1964 Helsinki declaration and its later amendments or comparable ethical standards.

### Informed consent

Informed consent was obtained from all individual participants included in the study.

## Results

### NAA/Cr ratios

A significant rise in the NAA/Cr levels in 5/12 analyzed voxels was observed in the following locations (Table 2): in the PFC on the left and right; in the right ventral ACG; in the left FCSWM; and in the right PCG (p = 0.002, p = 0.002, p < 0.001, p = 0.001 and p < 0.001, respectively). In the left dorsal ACG, the right IPS, and the left IPS, the increase trended toward significance (p = 0.019, p = 0.015 and p = 0.027, respectively).

**Table 2:** Longitudinal changes in NAA/Cr levels in named locations in the brain obtained on long-echo time MRS (p ≤ 0.004 significant, 0.05 > p > 0.004 trending toward significance).

NAA/Cr	N	Mean	SD	Min	Max	Percentiles			Z*	p*
						25^th^	50th Median	75^th^		
**Right prefrontal cortex**										
2011	19	1.636	0.229	1.21	2.16	1.480	1.690	1.800	−3.059	0.002
2016	19	2.017	0.330	1.54	2.69	1.750	2.010	2.230		
**Right ventral ACG**										
2011	19	1.402	0.252	0.83	1.91	1.220	1.350	1.600	−3.622	<0.001
2016	19	1.890	0.173	1.58	2.24	1.740	1.870	1.980		
**Left ventral ACG**										
2011	19	2.235	0.422	1.63	3.20	1.900	2.200	2.520	−0.644	0.520
2016	19	2.141	0.294	1.63	2.56	1.890	2.210	2.380		
**Left prefrontal cortex**										
2011	19	1.587	0.169	1.29	1.93	1.430	1.580	1.690	−3.159	0.002
2016	19	1.955	0.351	1.27	2.53	1.680	2.030	2.150		
**Right centrum semiovale WM**										
2011	19	2.514	0.503	1.74	3.52	2.020	2.490	2.880	−0.762	0.446
2016	19	2.392	0.539	1.62	3.53	1.990	2.150	2.790		
**Right dorsal ACG**										
2011	19	1.993	0.291	1.58	2.67	1.760	1.970	2.110	−1.503	0.133
2016	19	1.769	0.300	1.18	2.16	1.540	1.760	1.990		
**Left dorsal ACG**										
2011	19	1.483	0.282	0.72	1.92	1.370	1.530	1.680	−2.354	0.019
2016	19	1.781	0.380	1.07	2.67	1.530	1.720	2.020		
**Left centrum semiovale WM**										
2011	19	1.440	0.405	1.00	2.94	1.240	1.330	1.510	−3.341	0.001
2016	19	2.176	0.407	1.59	3.08	1.840	2.100	2.460		
**Right intraparietal sulcus**										
2011	19	2.057	0.350	1.62	2.89	1.780	1.980	2.200	−2.435	0.015
2016	19	2.376	0.380	1.59	3.01	2.160	2.400	2.690		
**Right PCG**										
2011	19	1.516	0.189	1.03	1.85	1.420	1.480	1.620	−3.824	<0.001
2016	19	1.947	0.236	1.62	2.45	1.740	1.920	2.140		
**Left PCG**										
2011	19	1.998	0.352	1.39	2.78	1.790	1.960	2.170	−0.806	0.420
2016	19	1.908	0.212	1.39	2.17	1.740	1.980	2.080		
**Left intraparietal sulcus**										
2011	19	1.902	0.316	1.47	2.85	1.670	1.910	2.010	−2.214	0.027
2016	19	2.163	0.329	1.65	2.93	1.940	2.030	2.420		

*Wilcoxon signed-rank test.

ACG-anterior cingulate gyrus, WM-white matter, PCG-posterior cingulate gyrus.

### Cho/Cr ratios

A significant increase in the Cho/Cr levels was detected in only one voxel, the right dorsal ACG (p = 0.001). However, in several additional voxels, we observed an increase in Cho/Cr levels that trended toward statistical significance in the left PFC (p = 0.013), in the left FCSWM (p = 0.005), and in the left (p = 0.007) and right IPS (p = 0.009) (Table 3).

**Table 3:** Longitudinal changes in Cho/Cr levels in named locations in the brain obtained on long-echo time MRS (p ≤ 0.004 significant, 0.05 > p > 0.004 trending toward significance).

Cho/Cr	N	Mean	SD	Min	Max	Percentiles			Z*	p*
						25th	50^th^ Median	75^th^		
**Right prefrontal cortex**										
2011	19	1.038	0.264	0.64	1.61	0.840	1.010	1.170	−0.698	0.485
2016	19	0.991	0.127	0.76	1.31	0.890	0.990	1.070		
**Right ventral ACG**										
2011	19	1.170	0.233	0.92	1.70	1.050	1.080	1.200	−1.787	0.074
2016	19	1.050	0.128	0.75	1.34	0.980	1.050	1.120		
**Left ventral frontal ACG**										
2011	19	1.038	0.174	0.79	1.49	0.890	1.040	1.160	−1.209	0.227
2016	19	1.167	0.237	0.89	1.71	1.020	1.060	1.320		
**Left prefrontal cortex**										
2011	19	0.954	0.0140	0.71	1.21	0.840	0.980	1.070	−2.476	0.013
2016	19	1.063	0.131	0.84	1.41	0.990	1.050	1.090		
**Right centrum semiovale WM**										
2011	19	0.860	0.187	0.60	1.27	0.680	0.870	1.030	−2.838	0.005
2016	19	1.049	0.197	0.69	1.40	0.920	1.030	1.180		
**Right dorsal ACG**										
2011	19	0.844	0.160	0.63	1.20	0.720	0.800	0.970	−3.200	0.001
2016	19	1.074	0.117	0.75	1.25	0.990	1.100	1.150		
**Left dorsal ACG**										
2011	19	1.065	0.146	0.87	1.39	0.940	1.050	1.160	−1.147	0.251
2016	19	1.092	0.075	0.93	1.22	1.030	1.100	1.150		
**Left centrum semiovale WM**										
2011	19	1.209	0.212	0.90	1.66	1.040	1.160	1.340	−1.610	0.107
2016	19	1.088	0.131	0.85	1.28	1.010	1.120	1.190		
**Right intraparietal sulcus**										
2011	19	0.901	0.122	0.68	1.21	0.810	0.890	0.990	−2.617	0.009
2016	19	1.052	0.125	0.89	1.35	0.960	1.030	1.170		
**Right PCG**										
2011	19	1.023	0.145	0.71	1.31	0.940	1.030	1.080	−0.262	0.794
2016	19	1.014	0.226	0.67	1.54	0.850	0.960	1.130		
**Left PCG**										
2011	19	0.793	0.218	0.49	1.27	0.620	0.790	0.910	−1.590	0.112
2016	19	0.911	0.143	0.72	1.17	0.790	0.870	1.050		
**Left intraparietal sulcus**										
2011	19	0.790	0.159	0.52	1.11	0.670	0.800	0.920	−2.677	0.007
2016	19	1.003	0.258	0.64	1.80	0.850	0.960	1.150		

*Wilcoxon signed-rank test.

ACG—anterior cingulate gyrus, WM—white matter, PCG—posterior cingulate gyrus.

### mI/Cr ratios

Longitudinal changes in mI/Cr were heterogeneous. We observed an increase in the mI/Cr level in the left ventral ACG (p = 0.002), the right dorsal ACG (p = 0.001), the right IPS (p < 0.001), and the right PCG (p = 0.001). In the left IPS (p = 0.029), the increase trended toward significance. In the left dorsal ACG, and in the right and left FCSWM, we confirmed a decline in mI/Cr (p = 0.033, p = 0.008 and p = 0.017, respectively), but this only trended toward significance (Table 4).

**Table 4:** Longitudinal changes in mI/Cr levels in named locations in the brain obtained on short-echo time MRS (p ≤ 0.004 significant, 0.05 > p > 0.004 trending toward significance).

mI/Cr	N	Mean	SD	Min	Max	Percentiles			Z*	p*
						25^th^	50^th^ Median	75th		
**Right prefrontal cortex**										
2011	19	0.483	0.184	0.22	0.87	0.320	0.480	0.650	−1.699	0.089
2016	19	0.708	0.448	0.32	1.91	0.440	0.560	0.750		
**Right ventral ACG**										
2011	19	0.674	0.238	0.34	1.19	0.520	0.650	0.840	−1.894	0.058
2016	19	0.553	0.151	0.28	0.90	0.430	0.560	0.650		
**Left ventral ACG**										
2011	19	0.465	0.148	0.20	0.66	0.320	0.460	0.580	−3.081	0.002
2016	19	0.601	0.109	0.32	0.77	0.570	0.620	0.660		
**Left prefrontal cortex**										
2011	19	0.587	0.121	0.39	0.80	0.510	0.600	0.650	−0.926	0.354
2016	19	0.649	0.160	0.43	1.05	0.530	0.640	0.790		
**Right centrum semiovale WM**										
2011	19	0.402	0.154	0.20	0.81	0.250	0.400	0.470	−2.657	0.008
2016	19	0.556	0.160	0.23	0.83	0.420	0.560	0.680		
**Right dorsal ACG**										
2011	19	0.391	0.147	0.21	0.89	0.310	0.350	0.430	−3.220	0.001
2016	19	0.614	0.122	0.30	0.78	0.550	0.590	0.720		
**Left dorsal ACG**										
2011	19	0.725	0.218	0.30	1.09	0.540	0.750	0.850	−2.134	0.033
2016	19	0.598	0.131	0.35	0.81	0.470	0.590	0.720		
**Left centrum semiovale WM**										
2011	19	0.712	0.180	0.31	1.09	0.610	0.710	0.830	−2.392	0.017
2016	19	0.575	0.209	0.15	1.20	0.460	0.570	0.620		
**Right intraparietal sulcus**										
2011	19	0.444	0.143	0.19	0.83	0.360	0.420	0.500	−3.543	<0.001
2016	19	0.650	0.146	0.34	0.85	0.540	0.680	0.770		
**Right PCG**										
2011	19	0.382	0.105	0.16	0.59	0.320	0.360	0.450	−3.315	0.001
2016	19	0.607	0.148	0.32	0.92	0.500	0.610	0.720		
**Left PCG**										
2011	19	0.415	0.101	0.27	0.61	0.350	0.390	0.470	−0.095	0.240
2016	19	0.418	0.103	0.22	0.67	0.370	0.400	0.420		
**Left intraparietal sulcus**										
2011	19	0.395	0.165	0.07	0.66	0.230	0.390	0.550	−2.179	0.029
2016	19	0.510	0.186	0.20	0.90	0.380	0.480	0.630		

*Wilcoxon signed-rank test.

ACG—anterior cingulate gyrus, WM—white matter, PCG—posterior cingulate gyrus.

### NAA/Cho ratios

In the right frontal ACG (p < 0.001), the left PFC (p < 0.001), the left FCSWM (p < 0.001), the right IPS (p = 0.001), and the right PCG (p = 0.001), we observed a significant increase in the NAA/Cho ratio, while, in the right PFC and the left PCG, the increase trended toward significance (p = 0.011 and p = 0.009, respectively). However, in the right FCSWM and the dorsal ACG on the same side, a significant decline in this ratio was observed (p = 0.001 and p = 0.001, respectively), as well as a decrease in the left PCG (p = 0.009) that trended toward significance.

### NAA/mI ratios

In the right ventral ACG, the left FCSWM, the right IPS, and the right PCG (p = 0.003, p < 0.001, p = 0.001 and p < 0.001, respectively), a significant increase in NAA/mI was observed. A decrease in the NAA/mI ratio was significant in the right FCSWM (p = 0.003) and in the right dorsal ACG that trended toward significance (p = 0.009).

### Neurocognitive assessment

In the five-year follow-up period, there was a trend toward a better performance for functions of delayed recall and recognition of verbal material (RAVLT A7, RAVLT recognition list A), for tests of visuospatial orientation abilities (ROCF), and for visuospatial span (WMS-R visual span: backward and forward span) (Table 5). The results of the other neurocognitive tests showed no significant longitudinal differences.

**Table 5:** Longitudinal trends in specific neuropsychological tests obtained on the Wilcoxon signed-rank test (statistical significance set at p < 0.05).

Test	N	Mean	SD	Min	Max	Percentiles			Z*	p*
						25th	50th Median	75^th^		
MMSE										
2011	19	27.36	2.014	24	30	25.00	28.00	29.00	−1.179	0.238
2016	19	28.36	2.335	24	30	27.00	29.00	30.00		
**TmtA**										
2011	19	37.27	14.51	15	64	25.00	35.00	52.00	−0.460	0.654
2016	19	36.09	16.85	17	70	23.00	33.00	46.00		
**TmtB**										
2011	19	54.18	26.17	22	113	36.00	42.00	73.00	−0.059	0.953
2016	19	54.45	26.61	20	98	34.00	53.00	87.00		
**RAVLT A1-A5**										
2011	19	49.00	7.823	38	59	42.00	47.00	58.00	−1.513	0.130
2016	19	44.64	9.811	32	62	34.00	46.00	50.00		
**RAVLT list A6**										
2011	19	10.00	3.493	5	15	8.00	9.00	14.00	−1.487	0.137
2016	19	8.36	3.472	2	15	6.00	9.00	10.00		
**RAVLT list A7**										
2011	19	7.45	3.012	3	14	5.00	8.00	9.00	−2.661	0.008
2016	19	10.09	2.737	7	15	7.00	10.00	12.00		
**RAVLT recognition list A**										
2011	19	10.91	2.212	6	14	10.00	11.00	13.00	−2.553	0.011
2016	19	13.64	2.063	9	15	12.00	15.00	15.00		
**Verbal fluency S**										
2011	19	11.80	4.76	5	20	8.50	11.00	14.50	−0.298	0.765
2016	19	10.91	4.66	4	18	8.00	10.00	16.00		
**Verbal fluency K**										
2011	19	12.30	4.06	4	18	10.5	12.00	15.5	−0.358	0.720
2016	19	11.45	4.72	6	20	7.00	11.00	15.00		
**Verbal fluency L**										
2011	19	10.80	3.53	6	18	7.75	10.5	12.5	−1.542	0.123
2016	19	8.82	4.81	3	21	6.00	7.00	11.00		
**Verbal fluency animals**										
2011	19	22.20	6.29	16	34	17.75	19	29	−1.368	0.171
2016	19	20.36	5.85	14	31	14.00	18.00	25.00		
**WCST-cat**										
2011	19	5.45	1.29	2	6	3.00	6.00	6.00	−0.756	0.450
2016	19	5.00	1.73	2	6	3.00	6.00	6.00		
**WCST-nos**										
2011	19	1.18	2.09	0	7	0.00	0.00	2.00	−1.289	0.197
2016	19	0.36	0.67	0	2	0.00	0.00	1.00		
**WCST-pers.err**.										
2011	19	12.55	10.32	3	38	5.00	9.00	19.00	−0.153	0.878
2016	19	14.36	12.35	3	42	5.00	10.00	26.00		
**Digit span forward**										
2011	19	6.91	0.539	6	8	7.00	7.00	7.00	−1.586	0.113
2016	19	6.18	1.250	4	8	5.00	7.00	7.00		
**Digit span backward**										
2011	19	5.55	1.440	4	8	4.00	5.00	7.00	−0.997	0.319
2016	19	5.00	1.342	3	7	4.00	5.00	6.00		
**Visuospatial span forward**										
2011	19	5.36	0.924	4	7	5.00	5.00	6.00	−1.925	0.044
2016	19	6.18	1.250	4	8	5.00	6.00	7.00		
**Visuospatial span backward**										
2011	19	5.09	0.944	4	7	4.00	5.00	6.00	−1.978	0.040
2016	19	5.91	1.044	5	8	5.00	6.00	7.00		
**ROCF copy**										
2011	19	31.09	3.254	25.50	36.00	28.50	31.50	34.00	−2.456	0.014
2016	19	33.86	2.491	28.00	36.00	34.00	34.00	36.00		
BDI-II										
2011	19	8.45	6.039	2.00	21.00	3.00	8.00	11.00	−0.494	0.621
2016	19	8.18	6.385	2.00	18.00	2.00	5.00	14.00		

*Wilcoxon signed-rank test.

MMSE—Mini Mental Score Examination, TmtA—Trial making test A (time in seconds), Tmt B—Trial making test B (time in seconds), RAVLT—Rey auditory-verbal learning test, verbal fluency S, L, K, animals—Serbian adaptation of verbal fluency FAS test, WCST–Wisconsin card sorting test, cat–categories, pers. err. –perseverative errors, ROCF—Rey-Osterrieth complex figure, BDI-II –Beck Depression Inventory II.

## Discussion

Short-term longitudinal changes on MRS in the HIV-infected brain are well documented, and show a reduction of immune activation and inflammation in the brain parenchyma after the introduction of cART^1^. Despite the clear signs of neuroinflammation in the early phase of HIV infection, no signs of a neurodegenerative process have been observed^1–3^. Prior to the cART era, Chong *et al*. were the first to report a progressive reduction of the NAA level in HIV-positive subjects in a short follow-up period (three to eight months)^15^. Furthermore, based on later longitudinal studies, long-term control of both processes, neuroinflammation and consequent neurodegeneration, seemed to be suboptimal despite cART and good peripheral viral suppression^16^.

To the best of our knowledge, this is the first five-year follow-up longitudinal MR spectroscopic study on chronic, neurologically asymptomatic, aviremic HIV-positive patients, stable on cART (>one year at the baseline), with a normal MRI. According to the published data on neuropathogenesis of HIV infection and the suboptimal efficacy of cART in the brain, we expected to find signs of further, steady HIV-associated neuronal damage despite the reduction of an ongoing inflammatory process in patients with undetectable plasma viremia^1,3,4^.

Surprisingly, the results showed the expected, ongoing, low-level inflammation (reflected by a mI/Cr increase), and a clear long-term increase in NAA/Cr, suggesting the possibility of partial functional brain remodeling under cART.

**Figure 2:**
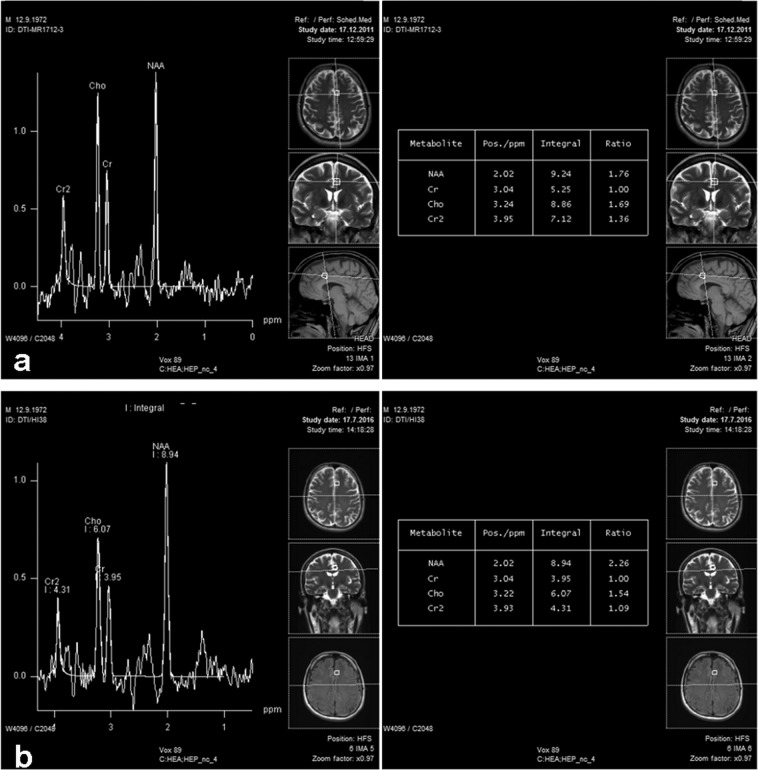
Follow-up long-echo time MR spectrum, performed in 2011 (**a**) and 2016 (**b**), with corresponding result tables, showing a progressive increase in NAA/Cr ratios.

The development of HIV-associated neurodegeneration might be more complex than merely a pure consequence of a continuous and uncontrolled inflammatory process in the brain parenchyma. An increase in the NAA/Cr level was observed in almost all analyzed voxels in our study (Fig. 2), with no areas where a decrease was evident. The NAA/Cr level is used as a relevant marker of neuronal injury and dysfunction, with a marked decrease in all pathological processes in the brain, which imply a loss of viable neurons, as well as disorders in normal neuronal functioning^17^. The increase in NAA is rather an unusual finding; transiently, it occurs after acute brain injury, whereas a stable increase has been reported in moyamoya disease, amyotrophic lateral sclerosis, and Wernicke encephalopathy^18,19^. In all these conditions, the NAA increase suggested brain plasticity and partial functional recovery. Cysique *et al*. recently described longitudinal decrease in NAA in PCG and frontal white matter in HIV-positive persons with HAND (both mild neurocognitive disorder and asymptomatic neurocognitive disorder). However, stable intact subjects showed NAA levels closest to healthy controls, implying that HIV-positive persons with normal cognition are less affected by neurocognitive decline and decline in NAA levels in these two locations^20^. In our study sample, we observed not only that these levels did not decrease in 5-year period, but in some locations, they increased, which might speak in favor of plasticity in the light of concomitant neuropsychological testing.

A recent study by Sanford *et al*., conducted as a two-year follow-up MR volumetric study, showed no significant volume loss or reduction of cortical thickness in aviremic, stable, HIV-positive patients on cART, compared to HIV-negative controls^21^. The results and the patient selection criteria are in accordance with our findings.

The fact that no statistically significant longitudinal changes in Cho/Cr were detected and statistical significance was reached in only one location (right dorsal ACG) in our study indicates good, long-term control of neuroinflammation under cART. In recent studies in chronic HIV-infection, a progressive decline in Cho/Cr was observed over time and explained as a probable reflection of tissue atrophy and a loss of brain parenchyma^3^. However, all these patients were symptomatic with severe neurocognitive disorders. This was not true for our patient population.

A transient increase in mI/Cr was observed in acute (up to eight weeks after transmission) and primary HIV infection (up to six months after transmission)^1^. Sailasuta and Young showed a reduction in mI/Cr soon after cART initiation; however, the levels of healthy controls were never reported^1,3^. Our finding of a dominant and stable increase in mI/Cr levels indicates a continuous microglial proliferation, obviously insufficiently controlled by highly penetrative cART. In addition, a neurotoxic effect from long-standing cART might also be implied, concordant with a recent study by Winston *et al*.^22^.

The increase in both Cho/Cr and mI/Cr ratios in our study may represent ongoing low-level inflammation and cell proliferation triggered by the presence of uneradicated HIV virus and its particles in the brain. This further suggests a suboptimal performance of cART in the brain, especially in the regions that have a significant number of microglial cells. This might explain the stable prevalence and incidence of milder forms of HIV-associated cognitive disorder in patients with cART.

Since the first studies on HIV-related brain injury, the diffuseness of the neurodegenerative process has been emphasized^23^. A rather global increase in NAA/Cr was observed in our five-year follow-up study. Nevertheless, the increase in mI/Cr was seen only in some locations (PFC, IPS, and FCSWM). This raises the question of whether certain brain regions are more susceptible to HIV injury due to complex pathways of HIV-associated neurodegeneration. Microglial cells are not equally distributed in the brain, with a variable density in the cerebral cortices, the brain stem, and the fiber bundles. Banati observed that persistent subtle microglial activity modulates neuronal functioning directly or indirectly, via astrocytic interaction. Microglial activation mobilizes functional compartments in which signaling molecules (neurokines, etc.) imply a more open cell-to-cell communication, not present in the healthy adult brain. These communications occur in neuronal pathways where this glial activation is under the influence of specific brain pathology, independent of disease etiology. This forms the framework for functional remodeling in the brain^24^.

During pathological process in the brain, the proliferation of microglia forms a network with regional differences that are not random, but functionally dependent^25^. Thus, microglial proliferation and membrane metabolism may be increased in those regions of the brain that constitute the brain circuits involved in attention and working memory, immediate and delayed recall, and visuospatial abilities, as a consequence of functional compartment remodeling in response to HIV-related chronic neuronal injury. The demonstrated increase in neurobiochemical markers could represent proliferation of radial glial cells that lead neuronal migration to the site of injury. The dynamics of neurobiochemical changes observed in the locations analyzed in this study were supported by the results obtained in some neurocognitive tests. Namely, we observed better performance for the functions of immediate and delayed recall, regulated mostly by the dorsal ACG and parts of the PFC^26,27^. Dysfunction in connections to the ACG results in apathy, impulsivity, and disinhibition - behavioral changes that occur in neurodegenerative diseases. Finally, we observed better achievement in visual attention and working memory, regulated by many networks in the brain, mainly PCG^28^. It comes as no surprise that previous studies on HIV-positive subjects confirmed the most prominent reduction in NAA/Cr levels exactly in this region. In light of these findings, a question could be raised about the functional remodeling of the brain pathways involved in cognition, predominantly in attention/working memory, visuospatial abilities, and delayed recall and recognition of verbal material. Sanford *et al*., in a recently published two-year follow-up study, showed better interval performance on several neurocognitive tasks, especially TmtA, concordant with our study. The authors commented on the result as a probable beneficial effect of the on-time introduction of cART and long-standing stable aviremia^21^.

### Limitations

The major limitation of the present study is the small number of participants, partially due to strict inclusion criteria. However, we performed a power analysis calculation for all three metabolite ratios. For NAA/Cr levels, the power analysis showed a satisfactory sample size (0.84–0.99), so that differences in NAA/Cr levels can be considered relevant despite a small sample size. The results of the power analysis for Cho/Cr and mI/Cr levels also showed a satisfactory sample size (0.98–0.99 and 0.86–0.99, respectively). Additional limitation is the partial volume effect of the cerebrospinal fluid, white matter or blood vessels) that can be present in some voxels that include mostly grey matter, such as voxels placed in cingulate gyrus. However, investigators that performed the MRS data processing controlled for this contamination and selected only spectra that best covered the ROIs with the grey matter. Finally, a significant issue might be the use of metabolite ratios instead of absolute values, given that the level of Cr is unstable in chronic HIV infected population, due to decreased uptake in the gut^29^, or changes in the brain synthesis of Cr.

## Conclusions

In summary, an unexpected longitudinal increase in NAA/Cr in neurologically asymptomatic HIV-positive patients on cART, observed on multivoxel MRS, followed by better performance on neurocognitive tests with regard to attention/working memory, delayed recall, recognition and visuospatial abilities, suggests functional remodeling of brain circuits in HIV-related neurodegeneration. Increased microglial proliferation and membrane metabolism might be implied in cell-to-cell communication and functional remodeling as a response to chronic neuronal injury. However, the latter may also reflect persistent inflammation and immune activation, probably due to the suboptimal efficacy of cART in the brain. Regional differences in of mI/Cr increases suggest that some brain regions are more prone to inflammation, either due to anatomical or neurophysiological features.
